# The Use of Surgiflo to Prevent Postoperative Bleeding in Paramedian Forehead Flap

**DOI:** 10.1097/GOX.0000000000002406

**Published:** 2019-09-10

**Authors:** Abeer Kalandar, Sarah Al-Youha, Richard Bendor-Samuel, Jason Williams

**Affiliations:** From the Division of Plastic and Reconstructive Surgery, Dalhousie University, Halifax, Nova Scotia, Canada.

## Sir,

The authors would like to present a safe and reliable method to stop pedicle bleeding in paramedian forehead flap nasal reconstruction. Postoperative bleeding from the flap pedicle can cause significant distress to the patient and frequently requires urgent physician intervention.^1^ Placement of pressure dressings, additional sutures, or using cautery, all carry risk of compromising the vascular supply to the flap. We propose the use of Surgiflo (Ethicon, Somerville, Mass.) over and around the flap pedicle to provide adequate hemostasis without threatening the flap vascular supply.

## TECHNIQUE

**Fig. 1.**
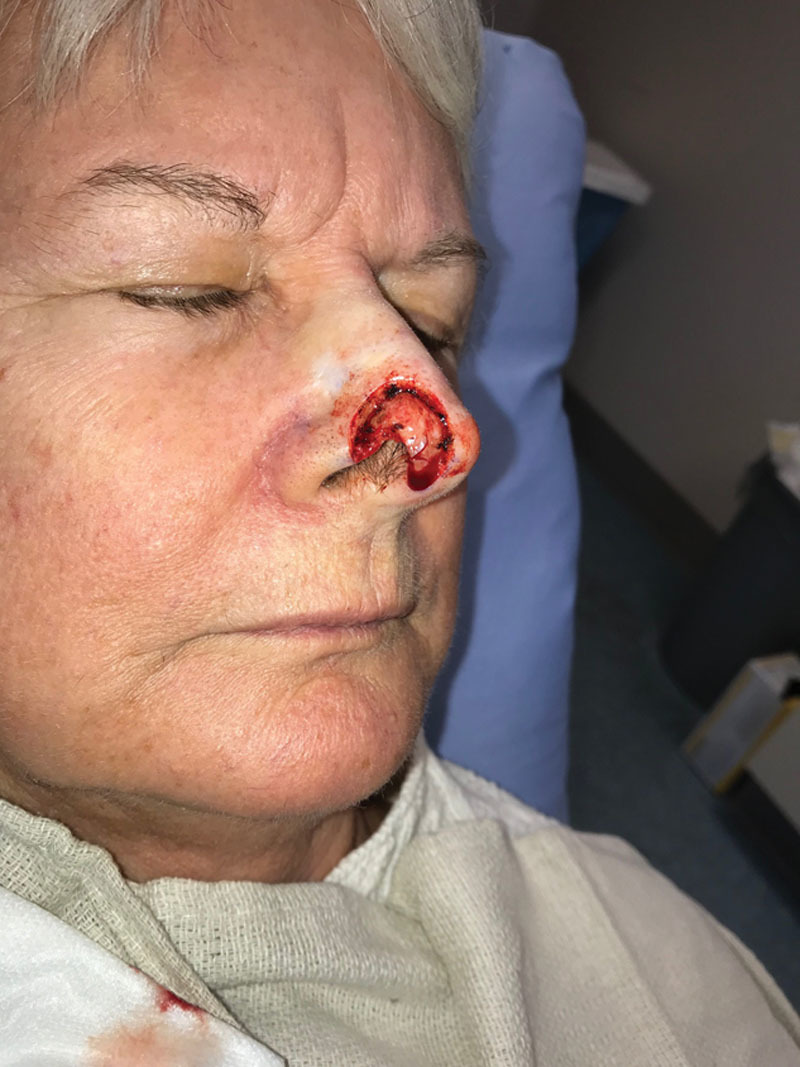
Preoperative picture showing nasal tip defect after Mohs surgery.

**Fig. 2.**
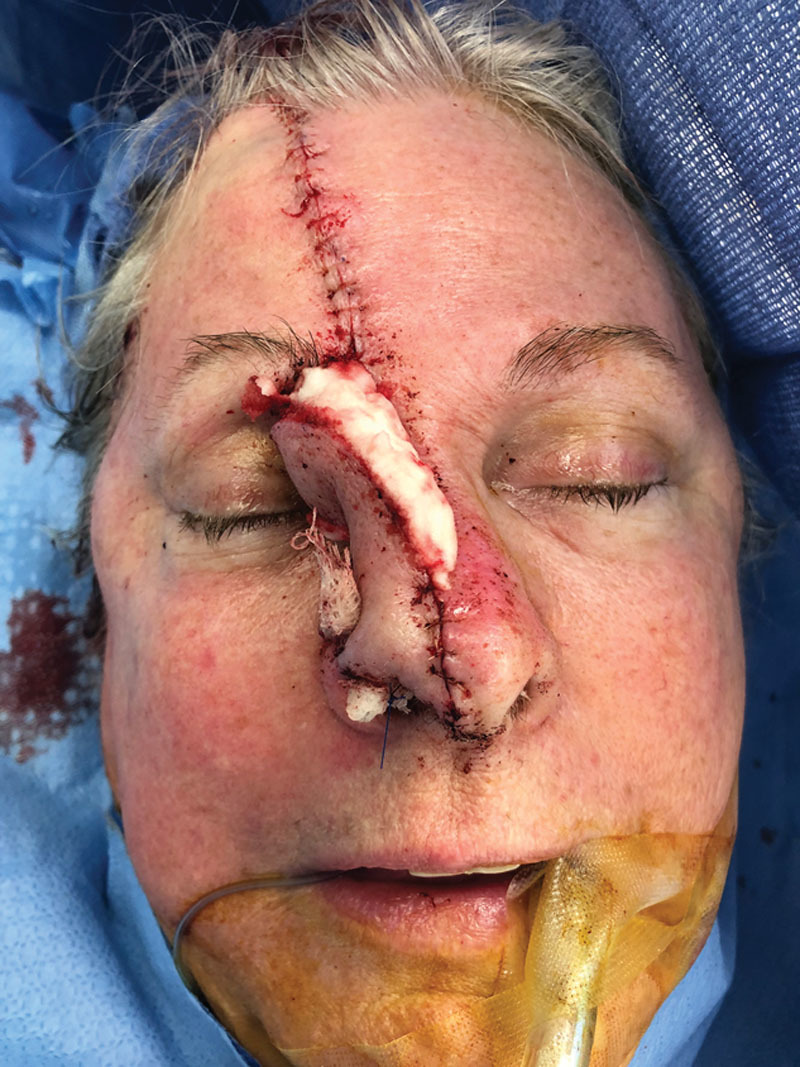
Immediate postoperative photograph after nasal tip reconstruction with paramedian forehead flap. Surgiflo was applied along the raw surface of the flap as shown.

We have used this product in our most recent cases and intend to use it for any future forehead flaps. The patient described here for illustration purposes presented with a nasal tip defect post-Mohs surgery (Fig. 1) and underwent paramedian forehead flap reconstruction. After flap elevation and inset, Surgiflo was mixed and applied following instructions provided by the manufacturer which can be found on the package insert and are easy to follow. Surgiflo was applied to the raw surface of the forehead flap to cover the entire area as shown in Figure 2. A sterile saline-soaked gauze was then applied over the Surgiflo and left for 2 minutes. Once the bleeding had ceased, the excess Surgiflo was irrigated away with normal saline.

The patient did not experience any significant bleeding and did not have any adverse events.

## DISCUSSION

Surgiflo is a thrombin-based hemostatic agent that has been demonstrated to be safe and reliable in achieving hemostasis in surgical wounds.^2^ To our knowledge, use of Surgiflo has not been reported in plastic and reconstructive surgery.

Several techniques have been used to stop pedicle bleeding in forehead flap reconstruction including split thickness skin grafts (STSGs) and oxidized cellulose gauze.^3^ Avoiding the harvest of STSG is favorable to both patients and surgeons. The disadvantages of using STSG include creating a second wound and risking prolonged healing of the donor site. Oxidized cellulose gauze causes excellent hemostasis, and the acidity promotes vasodilation at the flap pedicle. It is expensive and has reported complications including foreign body reactions and intramural hematomas.^4,5^ Standard dressings can be problematic because they do not aid in hemostasis directly and may become adherent to the wound due to blood absorption.

We believe that Surgiflo represents an ideal solution to the management of the raw surface of the pedicel in forehead flap nasal reconstruction. In our experience, it effectively stopped intraoperative and prevented postoperative pedicle bleeding without any adverse reactions.
